# The Use of Expectancy and Empathy When Communicating With Patients With Advanced Breast Cancer; an Observational Study of Clinician–Patient Consultations

**DOI:** 10.3389/fpsyt.2019.00464

**Published:** 2019-07-17

**Authors:** Liesbeth Mirjam van Vliet, Anneke L. Francke, Maartje C. Meijers, Janine Westendorp, Hinke Hoffstädt, Andrea W.M. Evers, Elsken van der Wall, Paul de Jong, Kaya J. Peerdeman, Jacqueline Stouthard, Sandra van Dulmen

**Affiliations:** ^1^Health, Medical and Neuropsychology Unit, Institute of Psychology, Leiden University, Leiden, Netherlands; ^2^Department of Communication, NIVEL, Netherlands Institute of Health Services Research, Utrecht, Netherlands; ^3^Leiden Institute for Brain and Cognition (LIBC), Leiden University, Leiden, Netherlands; ^4^Amsterdam Public Health Institute, Vrije Universiteit, Amsterdam, Netherlands; ^5^Department of Psychiatry, Leiden University Medical Center, Leiden, Netherlands; ^6^Department of Medical Oncology, University Medical Center Utrecht, Utrecht University, Utrecht, Netherlands; ^7^Department of Medical Oncology, St Antonius Hospital, Utrecht, Netherlands; ^8^Department of Medical Oncology, Netherlands Cancer Institute, Amsterdam, Netherlands; ^9^Department of Primary and Community Care, Radboud Institute for Health Sciences, Radboud University Medical Center, Nijmegen, Netherlands; ^10^Faculty of Health and Social Sciences, University of South-Eastern Norway, Drammen, Norway

**Keywords:** communication, placebo effects, nocebo effects, empathy, expectancy, cancer, palliative care, observational study

## Abstract

**Background:** Information provision about prognosis, treatments, and side-effects is important in advanced cancer, yet also associated with impaired patient well-being. To counter potential detrimental effects, communication strategies based on placebo and nocebo effect mechanisms might be promising to apply in daily practice. This study aimed to provide more insight into *how often* and *how* oncologists use expectancy and empathy expressions in consultations with patients with advanced breast cancer.

**Methods:** Forty-five consultations between oncologists and patients were audiotaped. To determine how often expectancy and empathy expressions were used, a coding scheme was created. Most consultations (*n* = 33) were coded and discussed by two coders, and the remaining 13 were coded by one coder. To determine how expectancy and empathy expressions were used, principles of inductive content analysis were followed.

**Results:** Discussed evaluation (i.e., scan) results were good (*n* = 26,58%) or uncertain (*n* = 12,27%) and less often bad (*n* = 7,15%). Uncertain expectations about prognosis, treatment outcomes, and side effects occurred in 13, 38, and 27 consultations (29%, 85%, and 56%), followed by negative expectations in 8, 26, and 28 consultations (18%, 58%, and 62%) and positive expectations in 6, 34, and 17 consultations (13%, 76%, and 38%). When oncologists provided expectancy expressions, they tapped into three different dimensions: relational, personal, and explicit. Positive expectations emphasized the doctor–patient relationship, while negative expectations focused on the severity of the illness, and uncertainty was characterized by a balance between (potential) negative outcomes and hope. Observed generic or specific empathy expressions were regularly provided, most frequently understanding (*n* = 29,64% of consultations), respecting (*n* = 17,38%), supporting (*n* = 16,36%), and exploring (*n* = 16,36%). A lack of empathy occurred less often and contained, among others, not responding to patients’ emotional concerns (*n* = 13,27% of consultations), interrupting (*n* = 7,16%), and an absence of understanding (*n* = 4,9%).

**Conclusion:** In consultations with mainly positive or uncertain medical outcomes, oncologists predominantly made use of uncertain expectations (*hope for the best, prepare for the worst*) and used several empathic behaviors. Replication studies, e.g., in these and other medical situations, are needed. Follow-up studies should test the effect of specific communication strategies on patient outcomes, to counter potential negative effects of information provision. Studies should focus on uncertain situations. Ultimately, specific placebo and nocebo effect-inspired communication strategies can be harnessed in clinical care to improve patient outcomes.

## Introduction

When faced with a serious disease such as advanced breast cancer, patients need information to understand what is going on and to plan for their future (1). Information about prognosis, treatment outcomes and plans, and benefits and risks of treatments are essential to provide optimal patient-centered care. Earlier data showed that patients having experienced adequate information about treatment benefits and risks experienced better person-centered care (2).

Despite its importance, information provision is by no means a “magic bullet” and also entails risks. There are several possible negative effects of information provision in advanced cancer. Explicit information about the incurability of a disease seems appreciated by most, but not all patients (3–5). Patients who are fully aware of their poor prognosis, are also the ones with the lowest reported quality of life and highest anxiety (6). It is known that providing information about side effects can increase their occurrence (7). A large study showed, for example, that breast cancer patients with relatively higher expectations of side effects are the ones experiencing the most side effects (8). While information provision is thus one of the cornerstones of communication (9), it can also lead to negative effects on patients’ well-being.

To counter any of these potential negative effects, communication strategies derived from placebo and nocebo mechanisms might be promising to apply in daily practice. Integrating the research worlds of communication and placebo effects is still in its infancy (10). Placebo effects can be seen as “all real biopsychological effects on patient outcomes that are not attributable to a medical-technical explanation” (11, 12). The most well-known mechanism *via* which placebo effects occur is the expectancy mechanism. There is ample evidence (mainly from experimental studies) that the use of positive expectations can influence clinical patients’ outcomes for the better (13, 14). For example, post-operative patients are known to experience less pain when pain medication is delivered in full view while verbally raising positive expectations about its effectiveness (15, 16). A second possible placebo effect mechanism affecting patient outcomes is the empathy mechanism, which is only mentioned by few scholars so far (10, 17, 18). From communication studies, we know that empathy is highly appreciated by patients (3, 19). From experimental studies in advanced breast cancer, we know that physician empathy is capable of reducing patients’ emotional distress, while increasing information recall (4, 20, 21).

It is, however, unclear if and how expectancy and empathy strategies are currently employed by clinicians when discussing prognosis, treatment outcomes, and side effects with patients with advanced cancer. The aim of this study is to provide more insight into *how often* and *how* oncologists use expectancy and empathy expressions in consultations with patients with advanced breast cancer. This study serves as a starting point for a research area aimed at creating more insight into possible beneficial placebo and nocebo effect inspired communication strategies. Future studies should test the effect of specific communication strategies on patient outcomes, before the most beneficial strategies can be harnessed in clinical care.

## Methods

### Design

We conducted a multi-center observational study of consultations between 12 oncologists and 45 patients with advanced breast cancer. Consultations were audiotaped, as audio observations provide more objective insights into communication behavior than self-reports. Data were collected between August and December 2018 at two Dutch city-based hospitals (one cancer-specific hospital and one general hospital).

### Ethical Approval

The study was evaluated by the Medical Ethical committee of the Netherlands Cancer Institute (NKI-AVL), which exempted the study from formal ethical approval. Both participating hospitals approved the conduct of the study in their representative hospitals. All subjects gave written informed consent in accordance with the Declaration of Helsinki.

### Sample

Initial consultations for patients with advanced breast cancer (i.e., the first time that patients would be informed that their disease is incurable) or follow-up visits in which evaluation results (i.e., scan results) would be discussed were included. It is likely that in these consultations, a detailed discussion of prognosis, treatment outcomes, and side effects would occur. The consultations had to include patients who were female, were ≥18 years of age, had advanced cancer in the sense that cure was no option anymore (according to the medical team), were not in the terminal phase of their disease, were cognitively able to provide consent and to complete a questionnaire, and who had command of the Dutch language.

### Recruitment

The medical team of the participating hospitals screened (mostly) weekly for eligible consultations and eligible patients. If there was too little time between identification of the consultation and the opportunity to recruit patients, eligible patients were not contacted. The remaining eligible patients were contacted by a member of the hospital team with a brief introduction of the study. The contact details of interested patients were transferred to the research team who explained the study in more detail *via* telephone contact with the eligible patient. More specifically, patients were informed that the study focused on communication between oncologists and patients, that one consultation would be audiotaped and that participants would have to complete both a pre-consultation question and a post-consultation questionnaire (only the post-consultation questionnaire assessing patient characteristics is included in this article, as this was a descriptive study). The research team did not mention the advanced stage of the disease. Preliminary oral consent was provided *via* telephone, after which patients were sent a written information letter *via* post or e-mail, and written consent was gathered by the research team immediately pre-consultation in the waiting area of the hospital. It was stressed that participation was voluntary and that patients could always withdraw their participation. Participating oncologists also provided consent for the consultations to be audiotaped.

### Sample Size

Being an audio-observation study of medical consultations (i.e., medical interviews) in which communication is explored in detail, data saturation was aimed for. Taken into account the variability in patients, oncologists, and consultations, we aimed for a somewhat larger sample of consultations than normally recommended (22) and aimed to include 35–40 consultations between patients and oncologists.

### Outcomes

#### Background Characteristics: Participants and Consultations

Patients’ sociodemographic characteristics (e.g., age, ethnicity, education) and disease characteristics (i.e., treatments currently receiving) were assessed post-consultation using a self-created questionnaire.

Characteristics of the consultation were assessed by the coding team. This included consultation time and whether the provided evaluation results (i.e., scan results) in the consultations were “good” (e.g., regression or stable disease), “uncertain” (e.g., clinical data from scan results and blood results are contradictory), or “bad” (e.g., disease progression). These criteria were determined in collaboration with the practicing oncologists who were part of the research and authorship team (EW, PJ, and JS). The core coding team (LV, MM, JW, and HH) determined together the category of each result.

#### Coding

To determine the occurrence of expectancy and empathy expressions, we created a coding scheme. This coding scheme was based on previous studies in the field of communication and placebo and nocebo effect research [expectancy references (23–28) and empathy references (4, 19–21, 29–35)], observations of other recorded consultations, and clinical and research expertise. See **Table 1** for a more detailed overview and explanation of the coding scheme.

**Table 1:** Coding scheme.

**Codes and examples of expectancy-expressions** *Code for each behavior how often it occurred and give the content (sentences) from which this became apparent.* *It is possible that an oncologist provided several remarks which e.g. illustrate that he/she is positive about the treatment outcomes. If that is the case, code each unique occurrence and provide the content for each occurrence.* *If there are two occurrences in one sentence, both are coded.* *Positive expectancy-expressions include expressions in which an oncologist expresses positive expectations about prognosis/treatment outcomes/side effects, negative expectancy-expressions include expressions in which an oncologists expresses negative expectations about prognosis/treatment outcomes/side effects, and neutral expectancy-expressions include expressions in which an oncologist expressed neither positive nor negative but neutral expectations about prognosis/treatment outcomes/side effects.*			
	**Positive (number + content)**	**Negative (number + content)**	**Neutral (number + content)**
**Prognosis** ***(referring to life expectancy/incurability)***	*“You are an active person, that will have a positive effect (on your life-expectancy, red)” (other taped consultation)*	*“You are not very fit anymore (talking about prognosis)” (other taped consultation)*	*“Your prognosis will also depend on your physical condition, how that will develop” (expert opinion)*
**Treatment outcomes** ***(referring to whether or not a treatment will work, and the possibility of (dis)continuation of treatment)***	*“I think this will work for you” (24)*	*“The problem is that there is little medication that is our go-to, there is not much better I can offer you” (25, 26)*	*“It is like a lottery; for some patients the treatment will work, for others it won’t. That’s all I can say unfortunately” (expert opinion)*
**Side effects** ***(mentioned with reassurance* → *positive*** ***might or might not happen* → *neutral*** ***mentioning side effect* → * negative)***	*“You should not believe all information on the internet. In my experience I have seen that around 80% of women respond very well with very little side effects” (expert opinion)*	*“You can also become much sicker because of the treatment” (other taped consultation)*	*“Fatigue can arise, but it might also not occur” (expert opinion)*
**Other**			
**Codes and examples of empathy expressions** *Code for each behavior how often it occurred and give the content (sentences) from which this became apparent.* *It is possible that an oncologist provided several remarks that, e.g., showed an interest in a person. If that is the case, code each unique occurrence and provide the content for each occurrence.* *If there are two occurrences in one sentence, both are coded.* *For coding of the behaviors, it is not necessary that a patient expressed an explicit cue/concern. If a cue or concern was expressed, which was not responded upon by the oncologist, this is coded as “missed opportunity”.*			
**Empathic behavior**	**Yes + number/content**	**No + number/content**	**Missed opportunities**
**NURSE**			
**a. Naming** ***(mentioning the occurring emotions explicitly)***	*“It sounds like you are worried” (*30*)*	*“I can see you are sad, but let’s talk about your medical situation” (expert opinion)*	
**b. Understanding** ***(showing understanding towards the emotions)***	*“I can’t imagine how difficult this news must be for you” (*31*)*	*“My experience is that most patients do not react to this news the way you do” (expert opinion)*	
**c. Respecting** ***(giving a compliment about emotion/response patient)***	*“I am very impressed with how well you’ve continued to care for your children during this long illness” (*30*)*	*“I think your response is a bit exaggerated” (expert opinion)*	
**d. Supporting** ***(stressing that a patient will be continuously cared for by oncologist/hospital)***	*“But whatever action we do take, and however that develops, we will continue to take good care of you. We will be with you all the way” (*4*)*	*“I will now refer you to the community care nurse. I will see you after the operation” (expert opinion)*	
**e. Exploring** ***(exploring of further emotions)***	*“We’ve just discussed a lot. Tell me more about what you are feeling right now” (*31*)*	*“I don’t have any time left unfortunately. There are more patients waiting. I will ask a nurse to contact you” (expert opinion)*	
**Showing interest in the patient and her feelings, not just the disease**	*“Would you appreciate it if I would speak to your children? With or without you, whatever you prefer” [expert opinion, based on (*19*)]*		
**Not interrupting the patient** ***(only code in case of ‘no)***			

For the expectancy expressions, the coding scheme addressed the number and content of oncologist-expressed positive, negative, or uncertain expectations regarding i) prognosis, ii) treatment outcomes, iii) side effects, and iv) others. This latter category was created to ensure we would not miss any expectancy expressions that could not be captured in our predefined categories. We did, however, not encounter any “other expectancy expressions”; hence, this is not further discussed in the Results section.

For the empathy expressions, the coding scheme addressed the number and content of the following oncologist-expressed empathic behaviors (irrespective of patients’ expressed emotional expression, called “cue” or “concern”) (36): i) NURSE (Naming, Understanding, Respecting, Supporting, Exploring) (30, 31); ii) showing interest in the patient and her feelings, not just the disease (19); iii) not interrupting the patient (only “negative” was coded); and iv) other. We coded both the occurrence of an empathic behavior as well as a non-empathic behavior. We created a third response category in case patients provided an emotional expression, which was not picked up by oncologists, labeling this a “missed opportunity for empathy” (37).

### Analyzing Process

The actual analyzing process consisted of several steps. We followed the Strengthening the Reporting of Observational Studies in Epidemiology (STROBE) Statement (38) and the Standards for Reporting Qualitative Research (SRQR) guideline (39), for the quantitative and qualitative part of the study, respectively.

*Step 1:* Patients’ background characteristics and consultations characteristics were analyzed using descriptive statistics.

*Step 2:* The consultations were coded to determine how often expectancy and empathy expressions were used by clinicians. All consultations were transcribed verbatim and personal identifiers were removed. First, the audiotapes of the consultations were listened to and the transcripts were read several times. Next, the abovementioned coding scheme (see **Table 1**) was applied and all specific positive/negative/uncertain expectancy expressions and empathic/non-empathic behaviors including the missed opportunities for empathy were copy-pasted from Word to a dedicated Excel template in which the specific behaviors were grouped together. In addition, how often all behaviors occurred per consultation was noted. Two investigators (MM and JW) independently coded 33 out of the 45 (73%) transcripts. All transcripts and coded segments were discussed and any discrepancies were resolved through discussion until a consensus was reached. The remaining 27% (*n* = 12) was coded by one investigator (JW). A third investigator (LV) coded all segments of a random 10% of the consultations (*n* = 4). Agreement between the investigators for all coded segments was 96.45% (136 out of 141 segments). Descriptive statistics were used to describe how often all expectancy and empathy expressions occurred per consultation. To facilitate analyses, Stata 14.0 was used.

*Step 3:* The expectancy- and empathy-coded text segments were used to determine how oncologists use these behaviors in consultations. To do so, all the coded segments that were grouped together were explored following the principles of inductive content analysis (40). First, in the preparation phase, the text was read several times, and two researchers (LV and JW or HH) independently wrote a memo for each subset of coded behavior, with most remarkable outcomes and sub-division of behaviors. These were discussed among the core researchers (LV, JW, MH, and MM). Next, in the organizing phase, text fragments belonging together were highlighted and codes were given. Emerging codes were grouped together under headings and compared to the entire dataset. In the final, reporting, phase, the final categories representing sub-forms of specific behaviors were determined. One researcher systematically coded all text (LV, communication/psychology background), while interim results were discussed among the research team (with a psychology, nursing, sociology, medicine, and communication background) to prevent one-sided interpretation of the data (41).

## Results

### Participants

All approached oncologists participated (*n* = 12). A total of 84 patients gave permission to be contacted by the research team. Of these, 19 gave no oral consent (they were not interested or found it too burdensome for the consultation to be audiotaped and/or to complete the questionnaires), 4 did not fulfill the inclusion criteria (e.g., they were scheduled for a check-up visit), 2 could not be reached by telephone, 10 encountered logistical problems preventing participation (e.g., there were 2 patients at the same time, the oncologist was too busy, or the consultation was cancelled), and 4 gave preliminary oral consent but withdrew their consent later. Lastly, for 2 patients who provided written consent, the audio-recordings failed. Background characteristics of the remaining 45 consenting participants are displayed in **Table 2**.

**Table 2:** Background characteristics of participants.

	Total (*n* = 41*)
	M (SD)
**Age**	57.18 (12.20) Range 31–84
	*n* (%)
**Marital status**	
Married	27 (66)
Single (including divorced, widowed)	14 (34
**Highest education** **^1^**	
Low	–
Intermediate 1	9 (22)
Intermediate 2	18 (44)
High	14 (34)
**Occupation**	
Paid job	10 (24)
Disabled/sick leave	14 (34)
Housewife	4 (10)
Retired	13 (32)
**Ethnicity**	
Dutch	35 (86)
Western immigrants	5 (12)
Non-Western immigrant	1 (2)
Treatments currently receiving**	
Chemotherapy	18 (44)
Radiotherapy	2 (5)
Hormone therapy	16 (39)
Immunotherapy	9 (22)
Operation	–
Targeted therapy	4 (9)
Symptom-oriented treatment	10 (24)
Tumor-oriented treatment possible, but refrained from	–
Tumor-oriented treatment impossible	1 (2)

^1^Low = primary education or less.

Intermediate 1 = lower secondary.

Intermediate 2 = upper secondary.

High = tertiary.

*Out of the 45 participating women, 41 completed all questionnaires, data of the remaining 4 could not be retrieved.

**Women can receive several treatments, so this does not add up to 100%.

### Consultations

The consultation lasted, on average, 18.96 min (SD = 8.00; range = 4.43–34.83). All consultations were evaluative follow-up consultation in which evaluation results (i.e., scan results) were discussed. In 26 consultations (58%), good evaluation results were discussed; in 12 consultations (27%), uncertain evaluation results were discussed; and in 7 (15%), bad evaluation results were discussed. There were no disagreements within the coding theme when determining to which category a consultation belonged.

### Use of Expectancy Expressions

#### How Often Are Expectancy Expressions Used?

##### Positive Expectations

Positive expectations about prognosis were provided in 6 (13%) consultations, followed by positive expectations about side effects, which occurred in 17 (38%) consultations, while in most consultations (*n* = 34, 76%), positive expectations about treatment outcomes were provided. On average, positive expectations about prognosis and side effects occurred less than once per consultation while positive expectations about treatment outcomes occurred more than twice per consultation (see **Table 3**).

**Table 3:** The occurrence of expectancy expressions throughout the consultations.

	Positive expectations			Negative expectations			Uncertain expectations		
	*n* (%)	M (SD) range	Examples content	*n* (%)	M (SD) range	Examples content	*n* (%)	M (SD) range	Examples content
**Prognosis**	6 (13)	0.40 (1.25) 0–7	*“Yes, but wait. For the time being, you’re still around”*	8 (18)	0.40 (1.03) 0–4	*“Um, well that makes that I don’t think your prospect is very positive”*	13 (29)	0.8 (1.84) 0–8	*“For how long this is going to go well? I hope for a terribly long time. Can I predict it fully? No I don’t know. Every time it’s for me also a bit hoping that it’s OK.”*
**Treatment outcomes**	34 (76)	2.58 (2.30) 0–10	*“No, these numbers are not disturbing at all, those tumor markers. I sometimes see numbers of 5,000 or 10,000”*	26 (58)	1.78 (2.39) 0–11	*“Um, well yes, that test result does scare me a bit, because … well, what you see on the scan is, well, that is not going well”*	38 (84)	4.29 (4.27) 0–23	*There’s always a possibility that it’ll work or a possibility that it won’t (…).: “And then you’re back at the point of this uncertainty.”*
**Side effects**	17 (38)	0.80 (1.24) 0–4	*“And we’re finding a better balance with the side-effects”*	28 (62)	1.91 (2.37) 0–8)	*“Because for tiredness I have no miracle cure.”*	27 (56)	2.05 (2.84) 0–12	*“And some people don’t experience this (side effect, red) at all and others a bit or very much (…) but there is no way to test that beforehand.”*

n = number of consultation in which specific expectancy expression occurred.

(%) = percentage of consultations in which specific expectancy expression occurred.

M = mean number of specific expectancy expression per consultation.

SD = standard deviation of specific expectancy expression per consultation.

Range = Range of specific expectancy expression per consultation.

##### Negative Expectations

Negative expectations about prognosis were provided in 8 (18%) consultations, followed by negative expectations about treatment outcomes, which occurred in 26 (58%) consultations, while in 28 (62%) consultations, negative expectations about side effects were provided. On average, negative expectations about prognosis occurred less than once while negative expectations about treatment outcomes and side effects occurred almost twice per consultation (see **Table 3**).

##### Uncertain Expectations

Uncertain expectations about prognosis were provided in 13 (29%) consultations, followed by uncertain expectations about side effects, which occurred in 27 (56%) consultations, while in 38 (84%) consultations, uncertain expectations about treatment outcomes were provided. On average, uncertain expectations about prognosis occurred less than once, while uncertain outcomes about treatment outcomes occurred more than four times per consultation (see **Table 3**).

#### How Are Expectancy Expressions Used

When oncologists employed expectancy expressions, they tapped into three different dimensions: i) relational, ii) personal, and iii) explicit. The relational dimension refers to the extent to which expectations enhance the oncologist–patient relationship. The personal dimension refers to the extent to which expectations incorporate a personal reflection from oncologists. The explicit dimension refers to the extent to which expectations are made explicit. The different dimensions occur to various degrees within positive, negative, and uncertain expectations.

##### Positive Expectations

**Figure 1:**
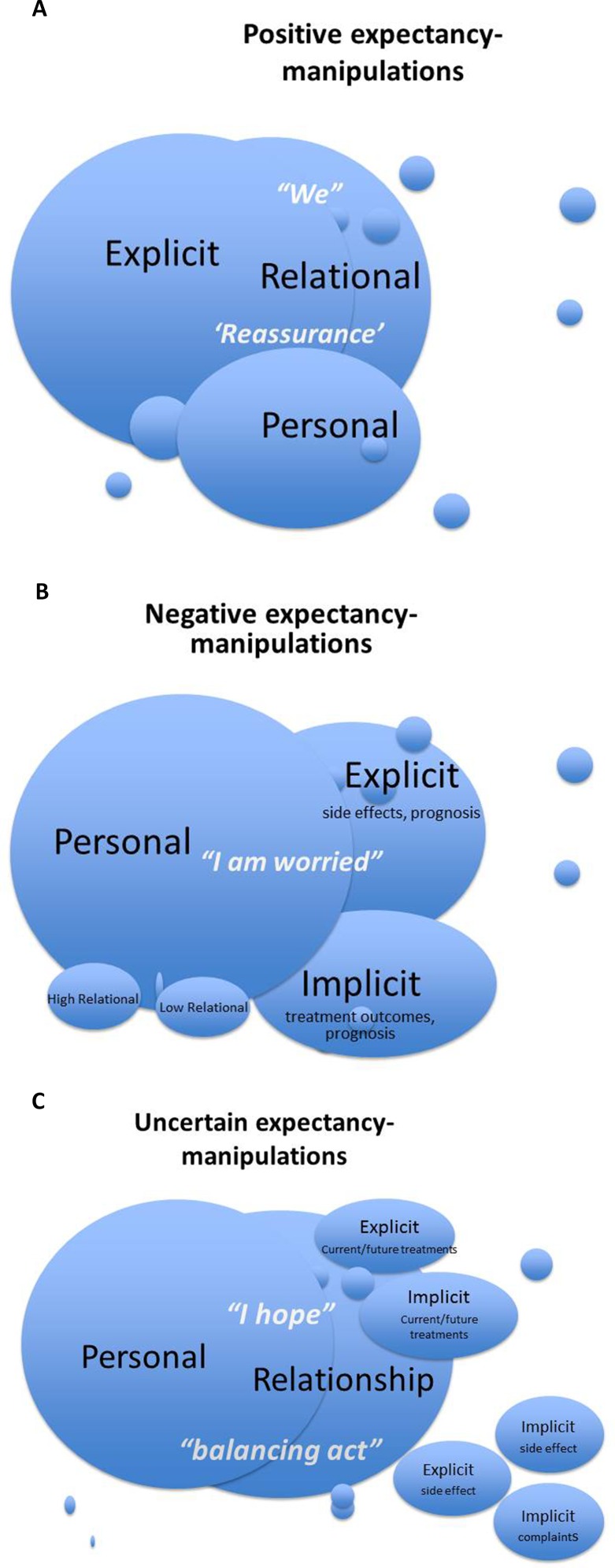
**(A)** Visual representation of the presence and overlap of the personal/relational/explicit dimensions of positive expectancy expressions. **(B)** Visual representation of the presence and overlap of the personal/relational/explicit dimensions of positive expectancy expressions. **(C)** Visual representation of the presence and overlap of the personal/relational/explicit dimensions of positive expectancy expressions.

Positive expectations were characterized by a high degree of—explicit—reassurance and thereby an emphasis on the doctor–patient relationship, while oncologists regularly referred to their personal thoughts and feelings. In **Figure 1A**, these different dimensions and their overlap are visually displayed. Patients were often reassured that there are still options available, that complaints are harmless, or that side effects will not be (or are not) too serious/burdensome. Such reassurance was frequently focused on very specific situations. Oncologists also regularly stressed their own thoughts and visions, which seemed to strengthen expressed positive expectations. Lastly, the doctor–patient partnership was often emphasized by referring to “we”.

*“I am not, I’m not worried about this at all. That scan is fine.”*

“With that reduced dose that (irritated mucous membranes, ed). will also get better”

“And we’re finding a better balance with the side effects”

Example of a quote where the personal, relational, and explicit dimensions come together:

*“Precisely, but just um looking into the far distance, I say yes, just carry on with it. Do we still have hormonal therapy as an alternative? Yes, if necessary we’ll use that. And if at a certain moment in time we are done with hormonal therapy, do we then still have something else? (…) Like chemo therapy? Yes. Even then there are some choices to be made and we’ll first and foremost have to make a choice that is then acceptable to you. (…) Do I have something good? Yes, I do. Is it acceptable to you? That is what we will talk about.”*

##### Negative Expectations

Negative expectations were characterized by a high degree of personal reflections, which seemed to strengthen a more or less explicit negative future vision. In **Figure 1B**, these different dimensions and their overlap are visually displayed. Oncologists expressed their own worries, about disease progression, a lack of treatment effects or side effects by which they seemed to emphasize the severity of the situation.

*“Do you want me to honestly tell you how um I think it’ll go? (…) Yes, I’m worried about you. Whether this will turn out well, because these blood counts, those blood platelets are suddenly so low.”*

“Because for tiredness I have no miracle cure.”

Such negative expressions varied in their level of explicitness, with treatment-related expectations often being expressed more implicitly than side-effect-related expectations, and with prognostic-related expectations being expressed both explicitly and implicitly.

*“For well, to be totally cured you have to, for that the various spots are actually too numerous.”*

“When all is said and done, the options I have are not infinite. Then it’ll grow and then it’ll get into your system and still further.”

With negative expectations, there was much less emphasis on relationship building. In the rare occasions the relationship dimension was tapped into, oncologists seemed to either emphasize or de-emphasize the clinician–patient relationship:

*“Yes, they are really nasty jabs. I have to admit that.”*

##### Uncertain Expectations

Uncertain expectations were characterized by an emphasis on what an oncologist hopes for, but cannot guarantee. While expressing such hopes, oncologists both focused on their own perceptions, making it personal, and on the positive relationship with patients. In **Figure 1C**, these different dimensions and their overlap are visually displayed.

*“For how long this is going to go well? I hope for a terribly long time. Can I predict it fully? No I don’t know. Every time it’s for me also a bit hoping that it’s OK.”*

Most importantly, uncertain expectations seemed to represent a balancing act. On the one hand, patients were being prepared for negative outcomes such as a future discontinuation of treatments or occurrence of problematic side effects. On the other hand, potential possibilities were mentioned, which were not presented as “magic bullets” but as a quest for a balance between treatment (intensity) and side effects.

*“So the first step is reducing the dose a bit and at a certain moment we’ll be putting in weeks of rest, with you doing two weeks followed by a week of no treatment. Um and doing so you hope that at a given time you’ll find a sort of stable situation that is doable for you, that you can get on with, doesn’t bother you too much yeah you’ll experience some bother, but something that you can get on with. If we should see that this causes problems, yeah well, then we’ll have to find the right balance, for that’s of course always what it is; the balance between side effect and effect.”*

Uncertain expectations about current and future treatment options and side effects were predominantly implicit in nature, but also sometimes more explicit (especially regarding treatment outcomes). They focused on (the source of) side effects and complaints that are currently present or might develop in the future, but also on the continuation of current and future treatments.

*“And some people don’t experience this (side effect, red) at all and others a bit or very much (…) but there is no way to test that beforehand.”*

“There’s always a possibility that it’ll work or a possibility that it won’t.’ Patient: ‘Umm mm.’ Oncologist: ‘And then you’re back at the point of this uncertainty.”

### Use of Empathy Expressions

#### Number of Expressions

##### Use of Empathy

All studied empathy expressions were displayed throughout the consultations, ranging from showing understanding of emotions in 29 (64%) consultations to the use of naming emotions in 4 (9%) consultations. The other empathy expressions occurred in around a third of consultations, e.g., respecting (*n* = 17, 38%), supporting (*n* = 16, 36%), exploring of patients’ emotions (*n* = 16, 36%), and showing interest in the patient (*n* = 13, 29%). On average, understanding remarks occurred more than twice per consultation, while all other statements occurred generally less than once per consultation (see **Table 4**).

**Table 4:** The occurrence of empathy expressions throughout the consultations.

Empathic behavior	Yes + number/content			No + number/content		
NURSE	*n*(%)	M (SD) Range	Examples content	*n*(%)	M (SD) range	Examples content
***a*. Naming** *(mentioning the occurring emotions explicitly)*	4 (9)	0.09 (0.29) 0–1	*“I can hear a sigh”*	–	–	
***b*. Understanding** *(showing understanding towards the emotions)*	29 (64)	2.27 (2.73) 0–13	*“Yes, I understand”*	4 (9)	0.09 (0.29) 0–1	*Patient: “Right. Um … is the therapy we’re using now enough to extend my life?”* *Oncologist: “Oh what a difficult question ha ha [loud laughter]”*
***c*. Respecting** *(giving a compliment about emotion/response patient)*	17 (38)	0.69 (1.08) 0–4	*“Well I agree. I think you are handling this very very well”*	–	–	
***d*. Supporting** *(stressing that a patient will be continuously cared for by oncologist/hospital)*	16 (36)	0.51 (1.01) 0–6	*“Or you can always give me a ring”*	1 (2)	0.02 (0.15) 0–1	*“I think that is really something for a psychologist”*
***e*. Exploring** *(exploring of further emotions)*	16 (36)	0.47 (0.73) 0–3	*Exploring specific: And um … What do you find stressful about it? Is it such a result or is it the Nivolumab itself?*	–	–	
**Showing interest in the patient and her feelings, not just the disease**	13 (29)	0.62 (1.23) 0–6	*“And how many years have you been married for?”*	1 (2)	0.02 (0.15) 0–1	*Patient: “I’ll handle this again. Well, yes the oldest son has Pfeiffer disease, so … Oncologist: Yes, you mentioned that. Patient: So, yes that … Oncologist: Let’s look at the blood pressure”*
**Not interrupting the patient** *(only code in case of “no”)*				7 (16)	0.2 (0.5) 0–2	*Patient: “Right, so it’s not as if you spinal column as one…. “ Oncologist: “It’s counted spot by spot.”*
**Missed opportunity** *(only code in the case of occurrence, which is thus negative)*	12 (27)	0.89 (2.36) 0–14	*Patient: “Aaahhh liver biopsy really is hell. But OK you’re right I’m not a wimp, but I really don’t like that, but well.” Oncologist: “No, well, right.”*			

n = number of consultations in which specific empathy expression occurred.

(%) = percentage of consultations in which specific empathy expression occurred.

M = mean number of specific empathy expression per consultation.

(SD) = standard deviation of specific empathy expression per consultation.

Range = range of number of specific empathy expression per consultation.

##### Lack of Empathy

Non-empathic behaviors were infrequently displayed throughout the consultation; interrupting the patient occurred in 7 (16%) consultations, followed by 4 (9%) consultations in which a lack of understanding occurred, while showing non-supporting statements or a lack of interest in the patient occurred in 1 consultation (2%). On average, negative behaviors occurred less than once per consultation (ranging from an average of 0.2 interruptions per consultation, to an average of 0.09 lack of showing understanding towards patient emotions per consultation). However, in more than a quarter of consultations (*n* = 12, 27%), oncologists failed to pick up on an emotional expression from a patient, which occurred, on average, 0.89 times per consultation (see **Table 4**).

#### How Empathy Expressions Are Used

##### Use of Empathy

When oncologists used empathy expressions, they used several manners to do so, which are closely aligned to the coding categories: NURSE (Naming, Understanding, Respecting, Supporting, Exploring) and showing interest in the person.

The most important distinction in empathy expressions referred to the level of specificity. Across the different NURSE categories, oncologists could either be generic in their level of expressed empathy, or, alternatively, could be specific. Specific empathic behaviors were characterized by referring to specific situations and emotions, or by referring to the individual.

Understanding generic: *“Yes, I understand.”*

Understanding specific: *“Yeah, so it’s really stressful, isn’t it.”*

Respecting generic: *“OK, that’s very good” (responding to a patient saying she will walk the dog on the beach).*

Respecting specific: *“What an extraordinary person you are, aren’t you.”*

Exploring generic: *“For um, how um do you feel about it.”*

Exploring specific: *“And um … What do you find stressful about it? Is it such a result or is it the Nivolumab itself?”*

When providing support, both generic and more specific statements were made that either referred to the oncologist proactively offering support, or referred to the patient proactively needing to request support.

Proactive oncologist generic: *“Is there anything else I can do for you?”*

Proactive oncologist specific: *“You know what, I’ll give you a call tomorrow morning to see if things are getting a bit better.”*

Proactive patient generic: *“Oh, right. Or you can always give me a ring.”’*

Proactive patient specific: *“Um … hey, so give me a ring next week if you haven’t recovered from that flue yet.”*

Lastly, there were several ways in which oncologists showed an interest in the patient as a person. These included enquiring about holidays, patients’ loved ones, important days coming up, and non-cancer-related health problems.

*“OK, nice where are you going?”*

“And how many years have you been married for?”

##### Lack of Empathy

Although a lack of empathy did not frequently occur, there were a few occasions in which oncologists showed little understanding of patients’ emotions by talking or laughing over them.

Patient: *“And um … well, that vocal cord, so you’re saying I’d better see the ENT doctor.”* Oncologist: *“We could also wait for a bit.”*

Patient: *“Right. Um … is the therapy we’re using now enough to extend my life?”* Oncologist: *“Oh what a difficult question ha ha [loud laughter].”*

The one occasion in which there was little interest in the person occurred when an oncologist failed to enquire about an ill loved one.

Patient: *“I’ll handle this again. Well, yes the oldest son has Pfeiffer disease, so …* Oncologist: *Yes, you mentioned that. Patient: So, yes that …* Oncologist: *Let’s look at the blood pressure.”*

If patients were interrupted, this was mainly because oncologists seemed to complete their sentences.

Patient: *“Right, so it’s not as if you spinal column as one….”* Oncologist: *“It’s counted spot by spot.”*

Lastly, oncologists sometimes did not respond to patients’ emotional expressions.

Patient: *“Aaahhh liver biopsy really is hell. But OK you’re right I’m not a wimp, but I really don’t like that, but well.”* Oncologist: *“No, well, right.”*

## Discussion

In this observational study of consultations between oncologists and patients with advanced breast cancer, we aimed to get an insight into and create a better understanding on *how often* and *how* oncologists make use of expectancy and empathy expressions in clinical care. While there has been a recent interest in the placebo and nocebo effects of communication, and clinicians’ empathic responses to patients’ expressed cues and concerns have extensively been studied (see, e.g., Zimmermann et al., 2007) (42), to the best of our knowledge, this is the first study to objectively determine how clinicians use expectancy and empathy expressions in advanced clinical breast cancer care. We found that in our sample, consisting of consultations in which mainly positive or uncertain medical outcomes were discussed, oncologists predominantly expressed uncertain expectations. Provided expectations differed in the extent to which they had a relational, personal, and explicit dimension. When expressing positive expectations, the doctor–patient relationship was emphasized, negative expectations focused on the severity of the illness, and uncertain expectations were characterized by a balance between (potential) negative outcomes and hope. Moreover, oncologists displayed several generic and specific empathic behaviors, most frequently showing an understanding towards patients’ emotions. A lack of empathy was not common, but mainly included oncologists not responding to patients’ emotional expressions. In sum, although various placebo and nocebo effect-inspired communication strategies were observed, their generalizability and their effects on patient outcomes remain to be determined, especially in uncertain situations with inherent uncertain expectations.

Focusing on expectancy expressions, several of our results are noteworthy. First, most (*n* = 26, 58%) consultations contained a “good” medical outcome (i.e., scan results), but positive expectancy expressions did not occur more often than negative or uncertain expectations. It might be that oncologists in our sample were reluctant to express—overtly—positive expectations in the context of advanced cancer, as patients are known to already often hold unrealistic expectations about their disease and treatment aims (43–45). This contrasts results from a study among heart disease patients, in which clinicians were often overly positive (46). Indeed, oncologists place great importance on not offering false hopes (47). Although very understandable, by refraining from positive expectations, oncologists might miss out on the potential helpful effects of this communication strategy. Patients appreciate it when clinicians are optimistic (48) and stress what can be done when facing an incurable cancer diagnosis (3, 49). Moreover, outside of the area of (advanced) cancer, positive expectations have shown to influence patient outcomes such as pain (evaluations) [(14, 50) (van Vliet et al., submitted)] and symptom burden (48). While it is a prerequisite that such expectations are realistic in nature, our insights suggest that there might be an underused potential for stressing positive aspects when communicating with patients with advanced cancer.

A second important observation was that expectation expressions differed not only in content (positive, uncertain, and negative) but also in the dimensions of being relational, personal, and explicit. By reassuring patients of the positive nature of outcomes, or by stressing that they hope for positive outcomes, oncologists in our sample did not only provide information but also seem to build a relationship, two distinct core functions of medical consultations (9). The stressful nature of discussing bad news (50), such as a lack of further treatment options, might, for some oncologists, limit the ability for relationship-building when providing negative expectations. In these situations, the severity of the situation is emphasized by making use of the negative impact of self-referring (e.g., *“I am worried”*) in contrast to its optimistic impact when raising positive expectations (e.g., *“I am not worried at all”*). Interestingly, in a series of experimental studies aimed at helpful communication styles, all communication elements that led to positive effects made use of a personal account (e.g., *“I understand you’re worried. We will look together at the options”*) (4, 20, 21, 33) stressing the potential power of this dimension, also in the context of bad news. Lastly, the explicitness in which expectations were expressed varied widely, with more explicit expectations emphasizing an anticipation and implicit expectations characterizing uncertainty.

Uncertain situations seemed to be of critical importance and difficulty when raising expectations. In uncertain expectations, oncologists in our study made use of a balancing act in which they prepare patients for potential or certain negative outcomes, while simultaneously trying to offer some forms of perspective. In the literature, such an approach is called *“Hope for the best, prepare for the worst”* (51), illustrating a dual pathway followed in serious and uncertain illnesses. Previous studies have shown that patients differ in their preferences for how to handle the uncertainty of their advanced illness, with some wanting more explicit information than others (52). Clinicians, meanwhile, are reluctant towards and have difficulty in discussing clinician uncertainty (53, 54). We indeed found that the level of explicitness in particular varied widely when providing uncertain expectations, illustrating a lack of clear guidance on how to do so best. With treatment and care options in advanced cancer becoming increasingly complex, and targeted and personalized medicine options rapidly growing, there is a pressing need to develop more insight into how oncologists should best deal with uncertainty and provide expectations with an uncertain nature.

Focusing on empathy expressions, a more straightforward picture seemed to emerge compared to expectancy expressions. Oncologists made use of various forms of empathy, most frequently of showing understanding for patients’ emotions and complimenting patients on how they handle their disease. The importance of acknowledging the emotions of patients with advanced cancer has been stressed before (49). Noteworthy, empathic remarks varied widely in their level of specificity, e.g., *“That’s good”* compared to *“You have handled situation X very well”*. As patients value to be seen and treated as an individual person (19), also when faced with an incurable cancer diagnoses (49), one could expect that more specific expressions of empathy are most appreciated and beneficial. Although intuitively logical, there is a lack of empirical evidence on the effect of more generic or specific empathic remarks.

Interestingly, while most patient complaints in medical care are about clinician communication, as well as in advanced illnesses [e.g., Refs. (55–57)], in our study, we found that a lack of empathic communication did not often occur. There were, however, occasions in which patients’ cues and concerns were not picked up by clinicians. Previous studies have shown that this is not uncommon in clinical practice (42, 58). If clinicians, however, do respond to emotional expressions, this can lead to positive outcomes, such as a decrease in consultation time (42), and an increase in the amount of information patients recall (58). Thus, based on our results, there seems to be room for improving the extent to which clinicians respond to patients’ emotional expressions, leading to potentially positive effects.

### Limitations

Our study has limitations. Firstly, our participants might not be representative for the entire population of people with advanced breast cancer, as they were female, highly educated, almost completely with a Dutch or other Western European background, and mainly recruited in a specialized research-focused cancer hospital. Secondly, our analyses were based on transcripts and thus verbal communication, while non-verbal elements such as eye contact remained masked. Intonation was used in the first but not latter phases of the qualitative analyzing process, as we used the transcripts for the coding. Thirdly, as we focused on the communication within the 45 audiotaped consultations, we did not take into account the nested design of our study (expectancy and empathy expressions were clustered within consultations, which were clustered within oncologists, which were clustered within hospitals). The number of audiotaped consultations per oncologist ranged from 1 to 8, while 8 of the 12 participating oncologists were from the specialized hospital, implying that the communication from the oncologists with more audiotaped consultations and from the specialized hospital influenced our results more strongly. Fourthly, given our limited sample size, we did not explore differences in used manipulations between consultations with a good, bad, or uncertain medical outcome. Fifthly, we only included consultations in which test results were discussed as these were the only ones identified, which potentially limits the generalizability of our results to initial consultations. Sixthly, as the research area of the placebo effects of communication is still in development, we welcomed the comment of one of the reviewers who wondered whether a comment as “that scan is fine” is a positive expectation and hope future discussions will help to clarify the criteria under study. Seventhly, although we did not observe other categories of expectancy expressions apart from our predefined categories, we cannot rule out that this is due to an implicit bias of the coding team, who all had a background in communication research. Our conceptualization was further hampered by a lack of a universally agreed conceptualization of expectancies [see, e.g., Laferton et al. (59) for a detailed overview]. Eighthly, we did not assess what patients’ information and communication preferences were. Lastly, although all approached oncologists participated, they might form a subgroup of clinicians particularly interested and competent in communication.

### Future Research

This study serves as a starting point for a research area aimed at creating more insight into possible beneficial placebo and nocebo effect-inspired communication strategies. The most pressing question our study does not answer is which specific forms of expectancy and empathy expressions are most promising in countering any negative effects of information provision and improving advanced cancer patients’ outcomes. Moreover, there is a need for a better understanding into why oncologists use specific placebo and nocebo effect-inspired communication strategies and which strategies are most appreciated by patients. These questions need to be answered in follow-up studies. Ultimately, evidence-based expectancy and empathy expressions should be recommended for clinical use in advanced cancer. This specifically applies to expectancy expressions in uncertain situations, which seem to be most complex, and the effect of more generic or specific empathic behaviors. Additionally, replication studies within our and other medical and cultural contexts are needed, e.g., in other diseases of a chronic and often ultimately fatal nature, in non-Western countries, and with other participants such as men or patients with low health literacy. Furthermore, future observational studies should focus in more detail on the expressed manipulations, e.g., focus on differences between dyads, oncologists, and (specialized) hospitals; on differences between consultations discussing varying medical outcomes; and on sequential analyses of expressed manipulations. Such studies could also include other potential forms of expectations, such as regarding procedures or expectations regarding patient behavior (e.g., self-efficacy). Lastly, larger replication studies could also focus on the relation between consultation time and the use of positive expectancy and empathy expressions. In our sample, given the limited sample size, we explored this association, which did not seem to be present [except for the expression of positive expectations about side effects, and for showing understanding towards emotions (*p* < 0.01)].

### Conclusions

To conclude, our study illustrated that when discussing positive or uncertain medical outcomes in advanced breast cancer, oncologists predominantly made use of uncertain expectancy manipulations. When providing positive expectations, oncologists emphasized the doctor–patient relationship, while negative expectations focused on the severity of the illness, and the area of uncertainty was characterized by a “hope for the best, prepare for the worst” approach. Moreover, empathy manipulations were generic or specific in nature and were dominated by oncologists showing an understanding towards patients’ emotions. A lack of empathy was uncommon, and mainly included oncologists not picking up on patients’ emotions. Follow-up studies should expand observational studies in this field, and focus on which communication strategies are most useful and influence patients’ outcomes for the better, to counter any potential negative effects of information provision. Such studies should focus especially on uncertain and complex medical situations, in which oncologists have to discuss uncertain expectations. Ultimately, specific placebo and nocebo effect-inspired communication strategies can be harnessed in clinical care to improve patient outcomes.

## Data Availability

The datasets for this manuscript are not publicly available because of ethical constraints. Requests to access the datasets should be directed to Liesbeth van Vliet, l.vanvliet@nivel.nl/l.m.van.vliet@fsw.leidenuniv.nl.

## Ethics Statement

The study was evaluated by the Medical Ethical committee of the Netherlands Cancer Institute (NKI-AVL), which exempted the study from formal ethical approval. Both participating hospitals approved the conduct of the study in their representative hospitals. All subjects gave written informed consent in accordance with the Declaration of Helsinki.

## Author Contributions

LV: conceptualization, methodology, data collection, data analyses, writing—original draft. AF: conceptualization, methodology, data analyses, writing—review and editing. MM: methodology, data collection, data analyses, writing—review and editing. JW: methodology, data collection, data analyses, writing—review and editing. HH: methodology, data collection, data analyses, writing—review and editing. AE: data analyses, writing—review and editing. EW: data analyses, writing—review and editing. PJ: methodology, data collection, data analyses, writing—review and editing. KP: methodology, data analyses, writing—review and editing. JS: methodology, data collection, data analyses, writing—review and editing. SD: conceptualization, methodology, data analyses, writing—review and editing

## Funding

This study was funded by a Young Investigator Grant of the Dutch Cancer Society (number 10392) awarded to Liesbeth van Vliet.

## Conflict of Interest Statement

The authors declare that the research was conducted in the absence of any commercial or financial relationships that could be construed as a potential conflict of interest.
